# Proteomic profiling of the phosphoproteins in the rat thalamus, hippocampus and frontal lobe after propofol anesthesia

**DOI:** 10.1186/1471-2253-14-3

**Published:** 2014-01-10

**Authors:** Jing Tang, Qiong Xue, Hong Ding, Zaisheng Qin, Jinfang Xiao, Chunshui Lin, Youtan Liu, Tao Tao

**Affiliations:** 1 Department of Anesthesia, Nanfang Hospital, Southern Medical University, Guangzhou, Guangdong, China; 2 Department of Anesthesia, The First Affiliated Hospital of Zhengzhou University, Zhengzhou, Henan, China; 3 Department of Anesthesiology, The University of Hongkong Shenzhen Hospital, Shenzhen, China

**Keywords:** 2D-gel electrophoresis, Anesthesia, Phosphorylation, Propofol, Rats

## Abstract

**Background:**

Propofol is a safe and effective intravenous anesthetic that is widely used for the induction and maintenance of anesthesia during surgery. However, the mechanism by which propofol exerts its anesthetic effect remains unknown. The rapid onset of phosphorylation modifications coincides with that of propofol anesthesia.

**Methods:**

Propofol-anesthetized rat models were built and phosphorylated proteins in the thalamus, hippocampus and frontal lobe were enriched the to analyze the changes in these phosphoproteins after propofol anesthesia.

**Results:**

Sixteen of these phosphoprotein spots were successfully identified using MALDI-TOF MS and a subsequent comparative sequence search in the Mascot database. Of these proteins, keratin 18 and the tubulin 2c chain are cytoskeletal proteins; keratin 18 and gelsolin are relevant to alcohol drowsiness. Based on Western blot analysis, we also confirmed that the phosphorylation of these proteins is directly induced by propofol, indicating that propofol anesthesia may be relevant to cytoskeletal proteins and alcohol drowsiness.

**Conclusions:**

These identified propofol-induced phosphorylations of proteins provide meaningful contributions for further studying the anesthetic mechanism of propofol.

## Background

Propofol is widely used in medical procedures such as gastrointestinal endoscopy in outpatient clinics [1], pediatric MRI examinations [2] and pediatric radiotherapy [3] because of its rapid onset, controllable delivery and rapid recovery. Because of its various advantages and wide range of applications, the mechanism of propofol as a general anesthetic has been the focus of increasing scientific research and much attention from anesthesiologists. Nevertheless, the specific mechanism remains unclear.

Thus far, anesthetic drugs have been known to exert their effect mainly by regulating both the synaptic transmission of key parts of the central nervous system and ion channels in the membrane [4]. Both the neurotransmitters that play an important role in synaptic transmission and ion channels are mostly proteins. Protein modifications, especially phosphorylation and dephosphorylation, play key roles in various cellular functions, such as cell differentiation [5], cell growth and apoptosis [6]. Kondratyuk et al. [7] confirmed that depolarization can increase the phosphorylation of sodium channels in a study conducted in rat brain synaptosomes. In addition, abnormal phosphorylation can cause abnormal cellular activities. Studies suggested that abnormal phosphorylation of tau in brain tissue precedes the formation of neurofibrillary tangles in Alzheimer’s disease [8]. Furthermore, increased tau phosphorylation has been reported in animals subjected to isoflurane and desflurane inhalation, which may contribute to the short-term cognitive dysfunction following anesthetic administration [9,10]. Therefore, detecting the changes in phosphorylation after propofol infusion will be of great help in exploring the underlying mechanism of the general anesthetic action of propofol.

The brain is a highly interactive entity, in which a number of separate brain areas cooperate to execute biological functions [11]. The thalamus may be thought as a type of relay and is believed to act as the switchboard of information between a variety of subcortical areas and the cerebral cortex [12]. Additionally, functional brain imaging also confirmed that the thalamus is the key target for anesthetic action [13]. Studies have shown that the hippocampus is responsible for mental behaviors such as initial learning and memory as well as for conscious behavior [14]. Wei H et al. [15] reported that propofol affects LTD expression in hippocampal CA1 dendrites in rats, which was assumed to be the reason for propofol-induced learning and memory damage. The cerebral cortex is the final target of arousal systems, and the dorsolateral prefrontal cortex is one of most important parts of the cerebral cortex, participating in activities such as emotion recognition, voluntary movements and working memory [16] as well as the maintenance of the arousal state in mammals [17]. Thus, phosphorylated proteins in the thalamus, hippocampus and frontal lobes were extracted in an animal model induced by propofol anesthesia, and the proteins that were differentially expressed before and after anesthesia were identified using two-dimensional electrophoresis and mass spectrometry in an attempt to uncover meaningful clues about the anesthetic mechanism of propofol.

## Methods

### Experimental animals and treatment

Forty-eight male Sprague–Dawley (SD) rats (180–220 g) were randomly separated into two groups: the control group (C group, n = 24) and the propofol group (P group, n = 24). To decrease the individual difference, every 4 rats were divided into a sub-group and tissues from the same sub-group were mixed together for one gel of 2D-electrophoresis. All animal procedures were approved and conducted in accordance with the guidelines for the care and use of animals of the ethics committee of Southern Medical University. Rats in the propofol group were assigned to receive a 10 mg/kg bolus injection of propofol (Astrazeneca, UK) delivered in 1 min and a continuous injection of 24 mg/kg/h propofol via a tail vein. The rats in the control group received an equivalent volume of 10% Intralipid (Sino-Sweden Pharmaceutical Corp., Ltd., China) via a tail vein. After 20 min, all rats were anesthetized with urethane and sacrificed immediately by decapitation. Three brain regions were collected and frozen in liquid nitrogen. The samples were stored at -80°C until further processing.

### Enrichment for phosphorylated proteins from tissues

Phosphoproteins from the thalamus, hippocampus and frontal lobe of the rats were enriched on a QIAGEN PhosphoProtein Purification column (QIAGEN, Valencia, CA) according to the manufacturer’s protocol. Briefly, 30 mg of tissue from the three brain regions were homogenized in 350 μl of lysis buffer containing 0.25% (w/v) CHAPS, protease/phosphatase inhibitors, and benzonase as described in the manufacturer’s protocol. The obtained phosphorylated proteins were purified with a 2D Clean-Up Kit (GE Healthcare). Next, the phosphoprotein yield was determined with a 2D QUANT Kit (GE Healthcare).

### Two-dimensional electrophoresis (2-DE)

An Immobiline Dry strip (pH 3–10 in the thalamus or pH 4–7 in the hippocampus and frontal lobe, 24 cm length, GE Healthcare) was rehydrated with 400 μg of phosphoprotein in 450 μl of rehydration buffer containing 4% CHAPS, 7 M urea, 2 M thiourea, 20 mM Trizma base, 65 mM DTT, 1% IPG buffer and 0.002% bromophenol blue for 14 hr at room temperature. Isoelectric focusing (IEF) was performed using the Ettan IPGphor 3 IEF System (GE Healthcare) for a total of 70 kVh. For the second dimension, SDS-PAGE was performed using an Ettan DALTsix Large Vertical system (Amersham, USA) according to the following procedures: 45 min at a constant power of 5 watts followed by 20 watts per gel until the bromophenol blue reached the bottom of the gel. The gels were then stained with 0.12% w/v Coomassie Brilliant Blue G250. The 2D gels were analyzed with the DeCyder software package (GE Healthcare, USA).

### MALDI-TOF MS identification and database searching

The peptide mixtures were identified on a Bruker Ultraflex III MALDI-TOF/TOF MS (Bruker Daltonics, Germany) operating in reflectron mode with 20 kV accelerating voltage and 23 kV reflecting voltage. Peptide mass fingerprints (PMFs) were searched against the SwissProt database using the program Mascot 2.1 (Matrix Science Ltd). The search parameters were as follows: trypsin digestion with one missed cleavage; carbamidomethyl modification of cysteine as a fixed modification; oxidation of methionine as a variable modification; peptide tolerance maximum of ±0.5 Da; MS/MS tolerance maximum of ±100 ppm; peptide charge of +1; and p < 0.05 for a local PMF search.

### Gene ontology analysis using GOMiner

Differentially expressed phosphoproteins were further classified using the GoMiner software [18] in combination with the GO database, which relies on a controlled vocabulary to describe a protein in terms of its subcellular localization, molecular function, or biological process.

### Western blot analysis

A total of 18 SD rats were randomly divided into three groups: the control group (C group), propofol anesthesia for 20 min group (P1 group) and propofol anesthesia 20 min followed by an arousal state for 1 h group (P2 group). The rats of the C and P groups were treated in the same manner as described above. The rats of the P2 group were exposed to the propofol anesthesia used in the P group for 20 min and were sacrificed 1 h post-anesthesia. Equal amounts of total protein (40 μg) and phosphoprotein (40 μg) were loaded and run on 12% SDS/polyacrylamide gels and transferred onto polyvinylidene difluoride membranes (PVDF) (Amersham Pharmacia Biotech, Piscataway, NJ). To detect keratin 18 and phospho-keratin 18, the membranes were probed with anti-cytokeratin 18 mouse monoclonal antibodies (1:500; Santa Cruz Biotechnology, Heidelberg, Germany). To detect gelsolin and phospho-gelsolin, the membranes were probed with anti-gelsolin mouse monoclonal antibodies (1:500; Santa Cruz Biotechnology, Heidelberg, Germany). To detect apolipoprotein E and phospho-apolipoprotein E, the membranes were probed with anti-apolipoprotein E goat polyclonal antibodies (1:500; Santa Cruz Biotechnology, Heidelberg, Germany). The immunoreactive bands were visualized using a Kodak 2000 M camera system (Eastman Kodak, Rochester, NY) according to the instructions of the manufacturer.

### Statistical analysis

All the data were tested for a normal distribution before statistical analysis, and the statistical analysis was performed using SPSS 13.0. The statistical significance of the difference among groups was evaluated using variance (ANOVA) followed by the Student-Newman-Keuls post hoc procedure. Significance was defined as P < 0.05.

## Results

### Quantitative comparison and identification of phosophoprotein spots on 2D gels

**Figure 1:**
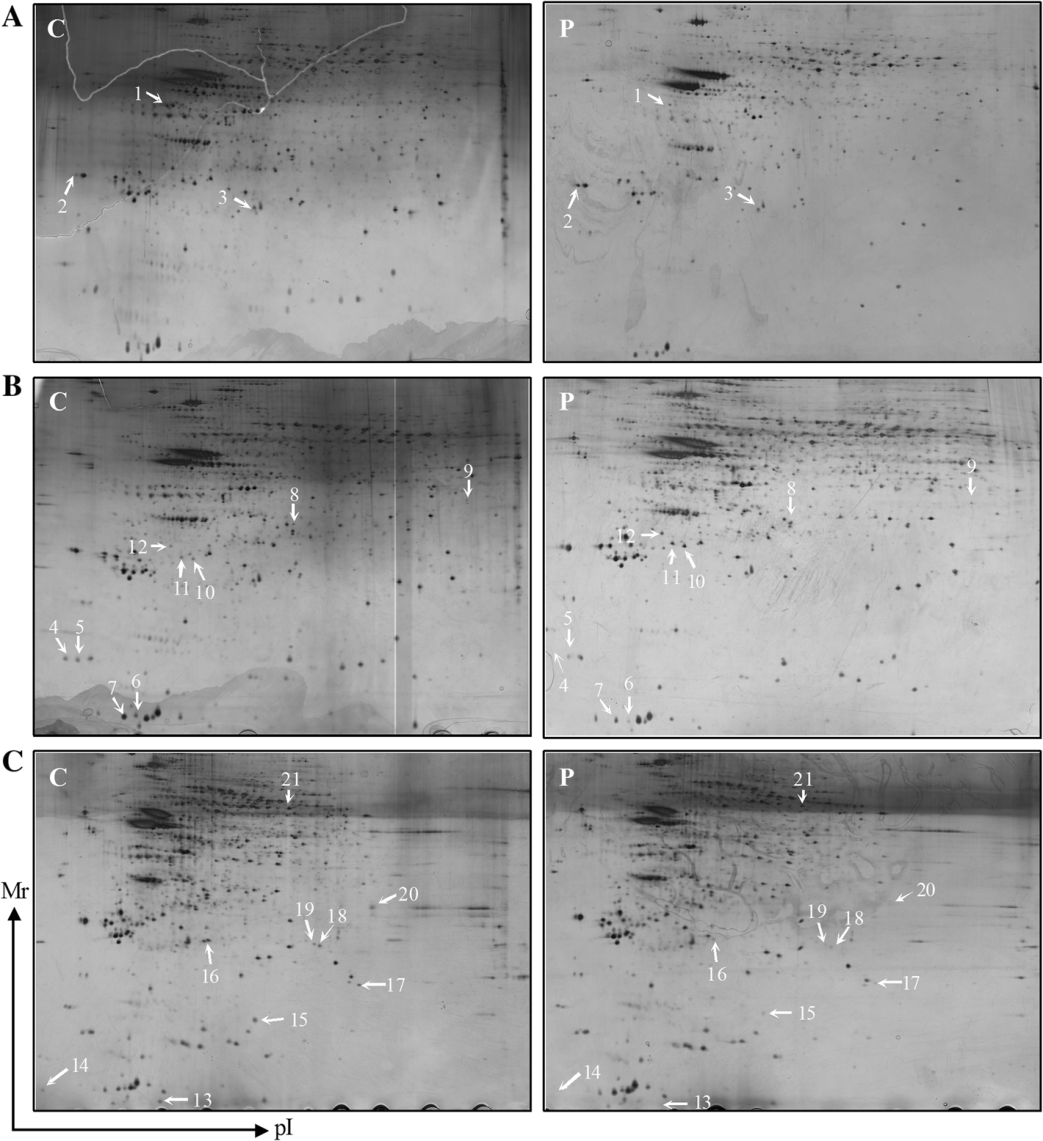
**Representative two-dimensional gels.** Control group (C), Propofol group (P). The phosphoprotein spots differentially expressed in the thalamus **(A)**, hippocampus **(B)** and frontal lobe **(C)**.

To determine the change in the phosphoprotein profiles of the thalamus, hippocampus and frontal lobe in response to propofol, gel-based comparative proteomic analyses were performed. As shown in Figure 1, 21 phosophoprotein spots were found to be significantly altered among the three brain regions. Sixteen of these phosphoprotein spots were successfully identified using MALDI-TOF MS and the subsequent comparative sequence search in the Mascot database (Table 1). Derived from the thalamus, gelsolin and hemoglobin were substantially up-regulated, but keratin 18 was down-regulated in the propofol group. Derived from the hippocampus, glutathione peroxidase 3, betaine-homocysteine S-methyltransferase 1, SET domain-containing protein 6 and cytochrome c oxidase subunit 5A were significantly overexpressed in the propofol group, while macrophage-capping protein, tubulin beta-2c chain and apolipoprotein E were expressed at a lower level than in the control group. The levels of delta-aminolevulinic acid dehydratase, 40S ribosomal protein SA, thioredoxin-like protein 1, ATP synthase subunit alpha, metastasis-associated protein MTA1 and actin, which were derived from the frontal lobe, were decreased in the propofol group.

**Table 1:** MALDI-TOF MS identification of differentially expressed protein

**Spot**^**a**^	**Protein name**	**Uniprot accession**	**Theoretical Mr (kDa)**^**b**^	**Total score**^**c**^	**Queries Mached**^**d**^	**P/C**^**e**^
1	Keratin 18	Q5BJY9	47.76	72	100%	↓
2	Gelsolin	Q68FP1	86.07	24	99.96%	↑
3	Hemoglobin	P02091	15.85	30	99.95%	↑
4	Macrophage-capping protein	Q6AYC4	28.80	148	100%	↓
6	Tubulin beta-2C chain	Q6P9T8	49.80	43	99.96%	↓
7	Apolipoprotein E	P02650	35.75	29	99.92%	↓
9	Glutathione peroxidase 3	P23764	25.27	87	100%	↑
10	Betaine-homocysteine S-methyltransferase 1	O96171	44.98	62	100%	↑
11	SET domain-containing protein 6	D3ZSK5	54.55	76	100%	↑
12	Cytochrome c oxidase subunit 5A	P11240	16.20	29	99.91%	↑
13	Delta-aminolevulinic acid dehydratase	P06214	36.03	33	99.95%	↓
15	40S ribosomal protein SA	P38983	42.91	150	100%	↓
16	Thioredoxin-like protein 1	Q920J4	32.25	156	100%	↓
18	ATP synthase subunit alpha	P31399	59.75	23	99.95%	↓
19	Metastasis-associated protein MTA1	Q62599	79.41	52	100%	↓
21	Actin, aortic smooth muscle	P62738	42.01	62	100%	↓

^a^The numbers indicate the spot positions in 2D gel as shown in Figure 1.

^b^Caculated from the database entry without any processing.

^c^By MALDI-TOF MS analysis.

^d^By MALDI-TOF MS analysis.

^e^P/C propofol group compared with control group.

### Gene ontology analysis of propofol-responsive phosphoproteins

The list of differentially expressed phosphoproteins was prepared for use in the GoMiner software in combination with the GO database. As seen in Tables 2, 3 and 4, the identified proteins were mainly distributed in the cytoplasm (12/16) and cytoskeleton (6/16), with functions in metabolism (10/16), regulation (8/16), stimulus response (6/16) and response to ethanol (5/16).

**Table 2:** The subcellular distribution of the identified proteins after anesthesia by propofol

**Subcellular**	**Protein name**
Cytoplasm	Keratin 18, gelsolin, MTA1, actin, Apolipoprotein E, M-capping protein, tubulin 2c chain, thioredoxin-like protein 1, Betaine-homocysteine S-methyltransferase 1, 40S ribosomal protein SA, ATP synthase subunit alpha, Delta-aminolevulinic acid dehydratase
Nucleus	keratin 18, M-capping protein, SET domain-containing protein 6, 40S ribosomal protein SA, MTA1
Cytoskeleton	keratin 18, gelsolin, tubulin 2c chain, M-capping protein, actin, Apolipoprotein E
Mitochondrion	Cytochrome c oxidase subunit 5A, ATP synthase subunit alpha

**Table 3:** The molecular function of the identified proteins after anesthesia by propofol

**Molecular function**	**Protein name**
Binding	MTA1, actin, Apolipoprotein E, ATP synthase subunit alpha, gelsolin, Macrophage-capping protein, Betaine-homocysteine S-methyltransferase 1, Hemoglobin, 40S ribosomal protein SA, Delta-aminolevulinic acid dehydratase
Catalytic	gelsolin, Glutathione peroxidase 3, tubulin 2c chain, Hemoglobin, Cytochrome c oxidase subunit 5A, Apolipoprotein E, SET domain-containing protein 6
Antioxidant	Glutathione peroxidase 3, Apolipoprotein E, Delta-aminolevulinic acid dehydratase, thioredoxin-like protein 1
Response to ethanol	gelsolin, Apolipoprotein E, Delta-aminolevulinic acid dehydratase, actin, Keratin 18

**Table 4:** The biological processes being participated in by the identified proteins after anesthesia by propofol

**Biological processes**	**Protein name**
Metabolic process	Keratin 18, Hemoglobin, ATP synthase subunit alpha, actin, thioredoxin-like protein 1, SET domain-containing protein 6, Betaine-homocysteine S-methyltransferase 1, Glutathione peroxidase 3, Apolipoprotein E, MTA1
Biological regulation	Keratin 18, gelsolin, Apolipoprotein E, Macrophage-capping protein, tubulin 2c chain, Betaine-homocysteine S-methyltransferase 1, SET domain-containing protein 6
Developmental process	Keratin 18, ATP synthase subunit alpha, tubulin 2c chain
Stimulus reponse	Keratin 18, gelsolin, thioredoxin-like protein 1, Delta-aminolevulinic acid dehydratase, Apolipoprotein E, SET domain-containing protein 6

### Western blot confirmation

**Figure 2:**
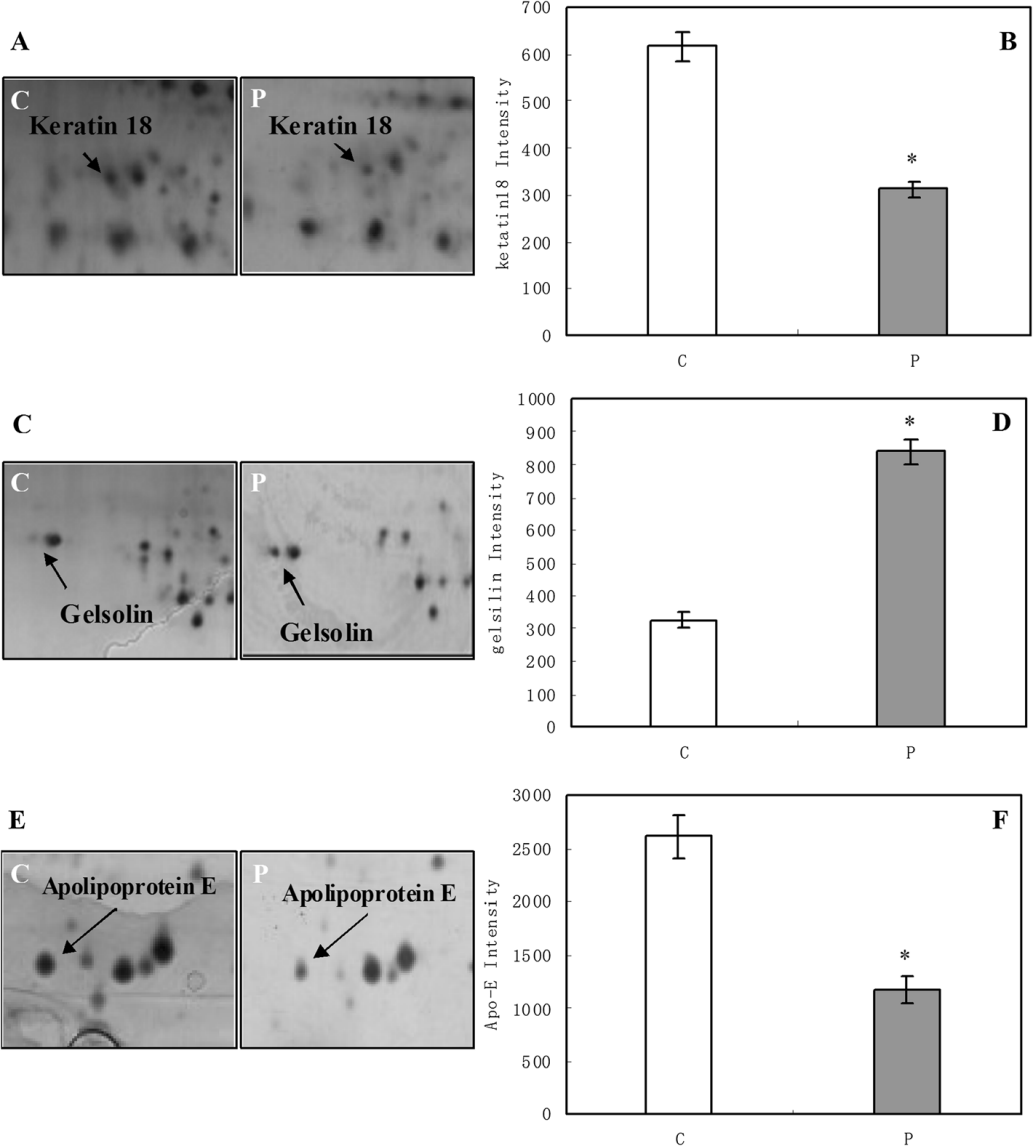
**The change in the amounts of phospho-keratin 18, phospho-gelsolin and phospho-apolipoprotein E in the propofol group.** Control group (C), Propofol group (P). The gels in the control and propofol groups have been enlarged to show the low expression of phospho-keratin 18 **(A)** and the high expression of phospho-gelsolin **(C)** and phospho-apolipoprotein E **(E)** in the propofol group. The corresponding gray intensity analysis of the 2D results **(B, D, F)**. The densitometric analysis of each protein was calculated from 6 different gels using PDQuest software. Each bar represents the mean ± SD (*P < 0.05, compared with the control group).

**Figure 3:**
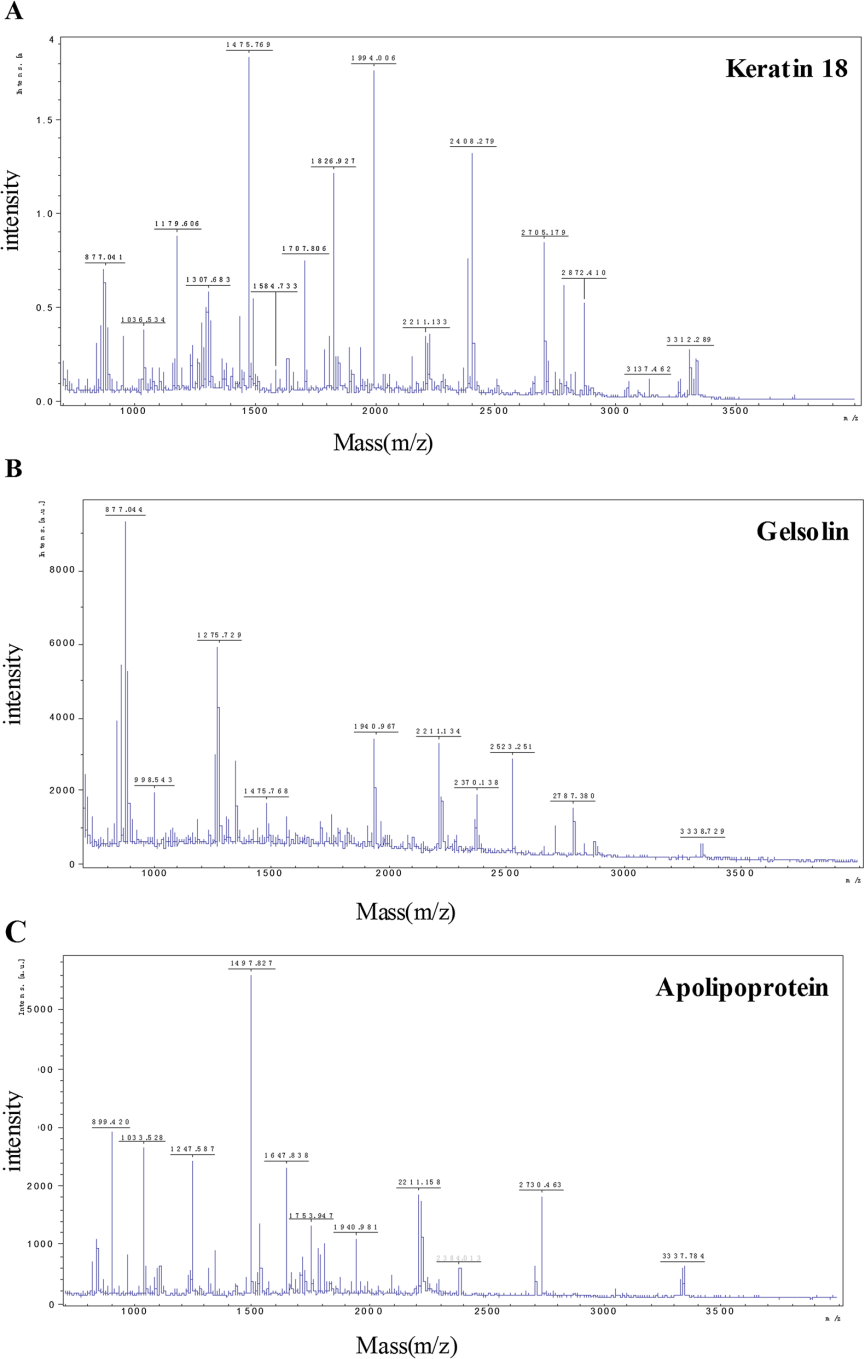
**Identification of keratin 18, gelsolin and apolipoprotein E using MALDI-TOF MS.** Mass spectrometry of in-gel trypsin digests of the proteins and analysis of the depicted peptide spectrum resulted in the identification of keratin 18 **(A)**, gelsolin **(B)** and apolipoprotein E **(C)**.

**Figure 4:**
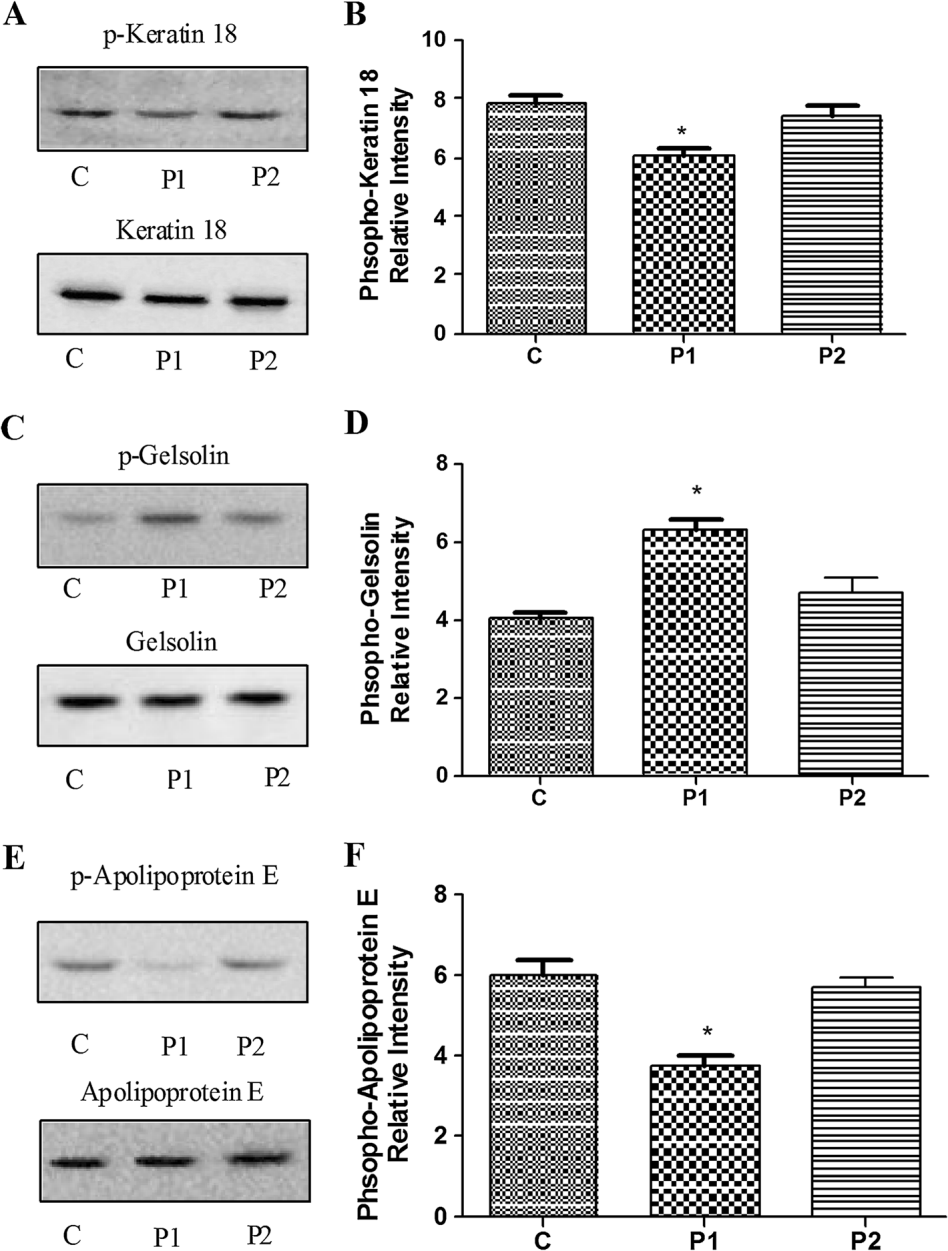
**Confirmation of the expression of phospho-keratin 18, phospho-gelsolin and phospho-apolipoprotein E with Western blot analysis.** Control group (C group), propofol anesthesia for 20 min group (P1 group) and propofol anesthesia 20 min followed by an arousal state for 1 h group (P2 group). Western blot analysis of phosphoprotein and total protein samples from the individual rats **(A, C, E)**. The corresponding gray intensity analysis of the Western blot results of the 3 groups **(B, D, F)**. Each bar represents the mean ± SD (*P < 0.05, n = 6, compared with the other 2 groups).

To verify the 2D results, keratin 18 and gelsolin from the thalamus and apolipoprotein E from the hippocampus were analyzed using Western blots. These proteins were selected because of interest in the components of the cytoskeleton. The corresponding differential keratin 18 expression patterns that were identified using 2D electrophoresis and the MALDI-TOF mass spectra are shown in Figure 2A-B and Figure 3A, respectively. To more rigorously study the effect of propofol on the protein phosphorylation, 18 rats were divided into three groups: the C, P1 and P2 groups. The amounts of phosphorylated and total keratin 18 were determined using Western blots. As shown in Figure 4A-B, the amount of total keratin 18 showed no significant difference among the three groups (P = 0.823). However, compared with the amount in the C and P2 groups, the level of phospho-keratin 18 in the P1 group decreased dramatically in accordance with our 2D gels results (P = 0.000). The corresponding differential gelsolin and apolipoprotein E expression patterns that were identified using 2D electrophoresis and the MALDI-TOF mass spectra are shown in Figure 2C-E and Figure 3B-C, respectively. As shown in Figure 4C-E, there were no observable changes in total gelsolin (P = 0.084) and total apolipoprotein E (P = 0.139). The amount of phospho-gelsolin in the P1 group significantly increased compared to the amount in the C and P2 groups (Figure 4C-D). (P = 0.000). The amount of phospho-apolipoprotein E in the P1 group significantly decreased compared to that in the C and P2 groups (Figure 4E-F) (P = 0.000). These results indicated that propofol can affect the extent of protein phosphorylation.

## Discussion

Although the anesthetic effect of propofol has been extensively studied by anesthesiologists, the molecular mechanism via which propofol exerts its anesthetic effect remains unknown. Research has demonstrated that decreasing the infusion speed of propofol could effectively decrease the side effects resulting from propofol anesthesia [19]. Larsson et al. [20] also found that the minimum amount of hemodynamic fluctuations and the most stable anesthetic effect occurred when propofol was delivered at a speed of 10 mg/kg/min. Because the anesthetized rats in this experiment were not subjected to any surgical procedures, the infusion speed could be set a lower level (24 mg/kg/h), with which a stable animal model was established.

Futter et al. [21] indicated that 7, 14 and 10 proteins spots were found to be differentially expressed in rat brain tissues 3 h, 24 h and 72 h after propofol anesthesia, respectively. Uniform differentially expressed protein profiles were noticed in the brain tissues of rats exposed to propofol or sevoflurane, and some proteins even showed a trend with the opposite change, indicating that the mechanism of action of inhaled anesthetics was not completely identical to that of intravenous anesthesia [22]. The insoluble protein profiles in the rat hippocampus were successfully identified, and their correlation with cognitive dysfunction after anesthesia was further explored by Xuena Zhang et al. [23]. These experimental results provided a new clue for explaining the molecular mechanisms of postoperative cognitive dysfunction after anesthesia. However, these studies examined total proteins, and protein synthesis is a time-consuming process. In contrast, protein phosphorylation is a fast and reversible process that is involved in a wide variety of crucial cellular activities [6], in which the transcription and synthesis of new proteins are not involved. In all post-translational modification, phosphorylation and dephosphorylation is the most important which regulate all activites of life including cellular signal transduction, cell differentiation, cell growth and apoptosis [5-7]. In addition, the rapid onset of phosphorylation modifications coincides with the onset of the propofol anesthesia. Therefore, the profile of phosphorylated proteins in the thalamus, hippocampus and frontal lobes was determined using proteomics in this study. Using mass spectrometry, sixteen proteins were found to be differentially expressed after propofol anesthesia, and the phosphorylation of keratin 18, gelsolin and apolipoprotein E was measured using Western blot to verify the 2D electrophoresis results.

The identified differentially expressed phosphorylated proteins were subjected to bioinformatics analysis. Six proteins, including keratin 18, gelsolin, tubulin 2c chain, macrophage-capping protein, actin and apolipoprotein E were identified. These proteins form cytoskeletal structures or participate in stabilizing the cytoskeletal structure. Additionally, the changing trends in the phosphorylation levels of the three proteins were verified using Western blots, suggesting that propofol anesthesia can cause changes in cytoskeletal proteins in brain tissue.

The cytoskeleton mainly consists of microtubules, microfilaments and intermediate filaments. The tubulin 2c chain is the main structural component of microtubules, and actin is the main constituent of microfilaments. In addition to maintaining the stability of cell shape and structure, cytoskeletal proteins also regulate various molecular activities such as intracellular transport [24], energy and information transfer [25], and signal transduction. Alterations in cytoskeletal architecture can result in changes in ion channels and account for the occurrence and development of brain-related disorders, which was first found in neurocytes. In 1981, Fukuda et al. [26] reported for the first time that cytoskeletal architecture disruption could inhibit the excitatory functions of sevrral central nervous systems (CNS) component. In 1988, Srinivasan et al., using the voltage-clamp technique [27], confirmed that the cytoskeleton was capable of modulating sodium channels in neurocytes. Propofol-induced amnesia may be related to the down-regulation of activity-regulated cytoskeleton-associated protein (Arc) in the hippocampus. In contrast, the Arc mRNA level does not change significantly, indicating that propofol may exert its function by affecting protein modification [28].

Microtubule-associated proteins (MAPs) bind to the tubulin subunits that form microtubules. Tau proteins are type II MAPs that are abundant in neurons in the central nervous system, and their phosphorylation state can be modulated by a specific set of phosphatases and phosphokinases, which play a vital role in maintaining neuronal function and development. Hyperphosphorylation of the tau protein is assumed to be involved in the neuropathogenesis of several types of dementia, such as Alzheimer’s disease and postoperative cognitive dysfunction [8]. Sevoflurane, isoflurane and propofol can induce tau hyperphosphorylation, which may account for the occurrence of postoperative cognitive dysfunction [10,29]. Reduced apolipoprotein E phosphorylation caused by anesthetic propofol was demonstrated and was later confirmed using Western blot in this experiment. Apolipoprotein E is synthesized and secreted predominantly by astrocytes and microglia in the brain, which participates in neuron repair after injury, dendritic growth and the maintenance of synaptic plasticity [30]. Apolipoprotein E also plays a vital role in the pathogenesis of Alzheimer’s disease, where apolipoprotein E is found in the amyloid plaques and neurofibrillary tangles characteristic of Alzheimer’s disease [31].

The deposition of monomers and polymers of hyperphosphorylated tau protein in the brain of a transgenic mice expressing apolipoprotein E4 (C112R) [32,33] suggested that apolipoprotein E affected tau phosphorylation. We speculate that the postoperative cognitive dysfunction induced by anesthetic propofol may result from alterations in serum apolipoprotein E levels. Nevertheless, the interwoven relationship between these factors and whether apolipoprotein E phosphorylation is involved in this process must be further explored.

Bioinformatics analysis indicated that gelsolin and keratin 18 exhibit responses to ethanol. Ethanol can weaken our body’s reactions to outside stimulation, which is similar to the behavioral changes that emerge after propofol anesthesia. Amino acid neurotransmitter receptors play an important role in alcohol dependence [34]. Ethanol is both a gamma-aminobutyric acid (GABA) receptor agonist and an N-methyl-D-aspartate (NMDA) receptor antagonist, which results in degenerative alterations of the nervous system during brain development by inhibiting ERK phosphorylation [35,36]. The effect of ethanol is similar to that of propofol on brain stem cell apoptosis during brain development. Glutamic acid (Glu) serves as the main neurotransmitter for the inputs and outputs as well as the intrinsic circuitry of the hippocampus, which appears to be the brain region that is most sensitive to alcohol damage [37] and is the target of the anesthetic effect of propofol. In summary, the targets of and transmitters in general anesthetic agents are similar to those of the effect of alcohol on the brain. Furthermore, studies have affirmed that ethanol was able to induce the dephosphorylation of keratin 18 in the liver and kidney [38]. In our study, keratin 18 was also dephosphorylated in the rat hippocampus following propofol administration compared with the controls. We speculated that the proteins responsive to ethanol may also contribute to the anesthetic effect of propofol. However, the specific mechanisms require further study.

## Conclusions

In conclusion, 16 differentially expressed phosphorylated proteins in the thalamus, hippocampus and frontal lobes were found using proteomics methods in this study. Additionally, bioinformatics were also used to analyze the common characteristics of the differentially expressed proteins to study the underlying mechanisms of the general anesthetic action. These experimental data will definitely provide meaningful references for the clarification of the mechanism of action of the general anesthetic propofol.

## Abbreviations

SD: Sprague–Dawley; IEF: Isoelectric focusing; PMFs: Peptide mass fingerprints; PVDF: Polyvinylidene difluoride membranes; CNS: Central nervous systems; Arc: Activity-regulated cytoskeleton-associated protein; MAPs: Microtubule-associated proteins; GABA: Gamma-aminobutyric acid; NMDA: N-methyl-D-aspartate; Glu: Glutamic acid.

## Competing interests

All authors declare that they have no competing interests.

## Authors’ contributions

JT, conducting of the study and editing language of the manuscript, approved the final manuscript and attested to the integrity of the original data and the analysis reported in this manuscript. QX, conducting of the study and preparing the manuscript, is the archival author and approved the final manuscript. HD, data collection, attested to the integrity of the original data and the analysis reported in this manuscript. ZQ, data analysis, approved the final manuscript. JX, data analysis, approved the final manuscript. CL, data analysis, approved the final manuscript. YL, study design, approved the final manuscript and attested to the integrity of the original data and the analysis reported in this manuscript. TT, study design, approved the final manuscript and attested to the integrity of the original data and the analysis reported in this manuscript. All authors read and approved the final manuscript.

## Pre-publication history

The pre-publication history for this paper can be accessed here:

http://www.biomedcentral.com/1471-2253/14/3/prepub
